# Association of the IL-10 and IL-18 polymorphisms with nasopharyngeal carcinoma risk

**DOI:** 10.3389/fonc.2025.1543182

**Published:** 2025-03-06

**Authors:** Xueru Chen, Ruibin Zhang, Hui Xie, Sha Li, Jincai Guo, Yan Wang

**Affiliations:** ^1^ Department of Pharmacy, Changsha Stomatological Hospital, Changsha, China; ^2^ School of Stomatology, Hunan University of Chinese Medicine, Changsha, China; ^3^ School of Pharmacy, Hunan University of Chinese Medicine, Changsha, China

**Keywords:** interleukin-10, interleukin-18, meta-analysis, nasopharyngeal carcinoma, polymorphism

## Abstract

**Objective:**

To evaluate the possible association of the cytokine polymorphisms with the risk of nasopharyngeal carcinoma (NPC).

**Methods:**

We performed a comprehensive search of electronic databases from PubMed, Web of Science, Embase, and CNKI. Articles related to the cytokine polymorphisms in patients with NPC and healthy controls from inception to 1 April 2024 were included. The results were analysed independently by two reviewers using RevMan 5.4 software. Summary odds ratio (OR) and 95% confidence interval (CI) were used to evaluate cancer risk.

**Results:**

Our results showed that IL-10 1082A>G showed a significant difference only in the Dominant model, but in the Asian population, a significant difference was shown in all models. IL-18 607C>A polymorphism showed significant differences in the Allele model, Heterozygote model, and Homozygote model. In addition, the IL-18 137G>C polymorphism showed significant differences in all models. No statistically significant association was found between IL-8 251A>T, IL-10 819T>C polymorphism, and the risk of NPC.

**Conclusion:**

Our meta-analysis results suggest that the IL-18 607C>A and IL-18 137G>C polymorphism are associated with the increased risk of NPC, and IL-10-1082 A/G polymorphism is associated with the increased risk of NPC in Asian populations.

## Introduction

Nasopharyngeal carcinoma (NPC) is an epithelial cancer that arises from the mucous membrane of the nasopharynx, often in the pharyngeal recess of the nasopharynx (1). NPC has a unique ethnic and geographic distribution, occurring in populations in East Asia, Southeast Asia, North Africa, and the Middle East (2). According to the latest statistics, the incidence of NPC is 1.5 cases per 100,000 person-years, and the incidence of males is about three times that of females (3). Studies have shown that a variety of factors, such as EBV infection, genetics, and environmental factors, can lead to NPC (4–6). Several clinical studies have found that about 25% of cancers are associated with inflammation (7–9), and inflammation is also a risk factor for NPC.

Cytokines are inflammatory factors, they are low molecular weight peptides that accumulate in the immune microenvironment, it affects the interaction and communication between cells (10). Cytokines promote various interactions between cancer cells and immune cells, are associated with various aspects of cancer development, and play a role in the carcinogenic or antitumor (11–13). Interleukin is a functional cytokine that is considered a major mediator of the inflammatory response. Its actions include the production of proteolytic enzymes, stimulation of lymphocytes, and enhancement of neutrophils (14). The interleukin-8 (IL-8) gene is located on human chromosome 4q13-q21, and the common gene polymorphism is -251A/T, which is closely related to the development of gastric cancer, breast cancer, colorectal cancer, and other cancers (15–17). The interleukin-10 (IL-10) gene is located between 1q31 and 1q32 on chromosome. There are three common polymorphisms in the promoter region of the gene: -1082 A/G, -819 T/C, and -592C/A. Studies have shown that these genetic polymorphisms are closely associated with the incidence and development of oral squamous cell carcinoma, breast cancer, and cutaneous malignant melanoma (18–21). The interleukin-18 (IL-18) gene is located on chromosome 11q22. The IL-18 gene promoter 607 C/A and 137 G/C polymorphisms are the two most common gene polymorphisms. Studies have shown that these gene polymorphisms are associated with the progression of NPC, prostate cancer, colorectal cancer, and other cancers (22–24).

Single nucleotide polymorphisms (SNPs) are DNA sequence polymorphisms caused by single nucleotide variations at the genomic level between individuals and are the most common genetic variants in the human genome (25). SNPs in any region of a gene can affect the protein structure or expression levels of the gene product, thereby altering an individual’s susceptibility to disease and affecting tumor development and progression (26, 27). Functional SNPs on cytokine coding genes can strongly induce malignant cell proliferation, enhance malignant transformation, and promote the development of NPC (28).

Currently, several studies have investigated the relationship between polymorphisms of inflammatory factors and the risk of NPC, which includes IL-8, IL-10, and IL-18, but the results are not completely consistent (29, 30). Additionally, there is no comprehensive meta-analysis on the relationship between the risk of NPC and polymorphisms in inflammatory factors. Therefore, we conducted a meta-analysis to better understand the relationship between cytokine polymorphisms and the risk of NPC.

## Methods

### Search strategy

Our search strategy involved the use of a combination of free-text terms and medical subject terms (MeSH terms). We comprehensively searched four key databases, namely PubMed, Embase, Web of Science, and CNKI, covering the period from their inception until April 1, 2024. Furthermore, we reviewed the reference lists of the included articles to identify additional relevant studies. The search strategy for PubMed was as follows:

1. “nasopharyngeal carcinoma”[MeSH Terms]
2. ”NPC”[Title/Abstract] OR “nasopharyngeal cancer”[Title/Abstract] OR “nasopharyngeal neoplasms”[Title/Abstract] OR “ UCNT “[Title/Abstract]
3. #1 OR #2
4. ”IL-18”[Title/Abstract] OR “interleukin-18”[Title/Abstract] OR “IL-4”[Title/Abstract] OR “interleukin-4”[Title/Abstract] OR “interleukin-12”[Title/Abstract] OR “interleukin-10”[Title/Abstract] OR “interleukin-18”[Title/Abstract] OR “IL-12”[Title/Abstract] OR “IL-8”[Title/Abstract] OR “IL-10”[Title/Abstract]
5. (variant [Title/Abstract]) OR (polymorphism [Title/Abstract])
6. #3 AND #4 AND 5

### Eligibility criteria

The inclusion criteria were as follows: 1) population-based case-control studies published as original articles; 2) investigating cytokines polymorphism and nasopharyngeal carcinoma;3) independent studies without repeated reports on the same population; 4) available detailed genotype data allowed to be calculated. The exclusion criteria were as follows: 1) meta-analyses, letters, reviews, or editorial articles; 2) absence of the proposed SNPs or complete data on genotypes; 3) no control population; 4) studies based on animals or cell lines.

### Data collection and quality assessment

Articles that did not meet the criteria were excluded according to the content of the article title and abstract. Subsequently, the full text of potentially relevant articles was carefully reviewed. After the full-text assessment, data were extracted from the article including the first author’s name, publication year, country, race, sex, age, number of included populations, genotyping method, allele counts in NPC cases, and controls, genotype distribution and The Hardy–Weinberg equilibrium (HWE). The screening and data extraction procedures were performed independently by JG and XC, and any disagreements were resolved by a third reviewer, HX.

Newcastle-Ottawa scale (NOS) was used to assess the risk of bias in the included studies. There were 11 items in total, with 1 point for each item. Studies with scores of 7 to 9, 5 to 6, and less than 5 were classified as high, moderate, and low quality, respectively.

### Statistical analysis

Statistical analysis was conducted using RevMan 5.4 software. Taking the IL-8 251 A/T polymorphism as an example, the Allele model (A vs T), Dominant model (AA + AT vs TT), Recessive model (AA vs the AT + TT), Heterozygote model (AT vs TT), and Homozygote model (AA vs TT) were calculated. Odds ratios (ORs) with 95% confidence intervals (95% CI) were used to evaluate the potential association of these functional SNPs with The risk of NPC, P < 0.05 was defined as significant. Heterogeneity was assessed by the chi-square test (α = 0.1) and the inconsistency index statistic (I^2^). If no heterogeneity was observed (P > 0.1, I^2^ ≤ 50%), the fixed-effect model was selected for meta-analysis. Conversely, if heterogeneity was observed (P ≤ 0.1, I^2^ > 50%), further investigation was conducted to identify the potential sources of clinical heterogeneity. Subsequently, a random-effects model was employed for the meta-analysis.

## Result

### Literature search

**Figure 1:**
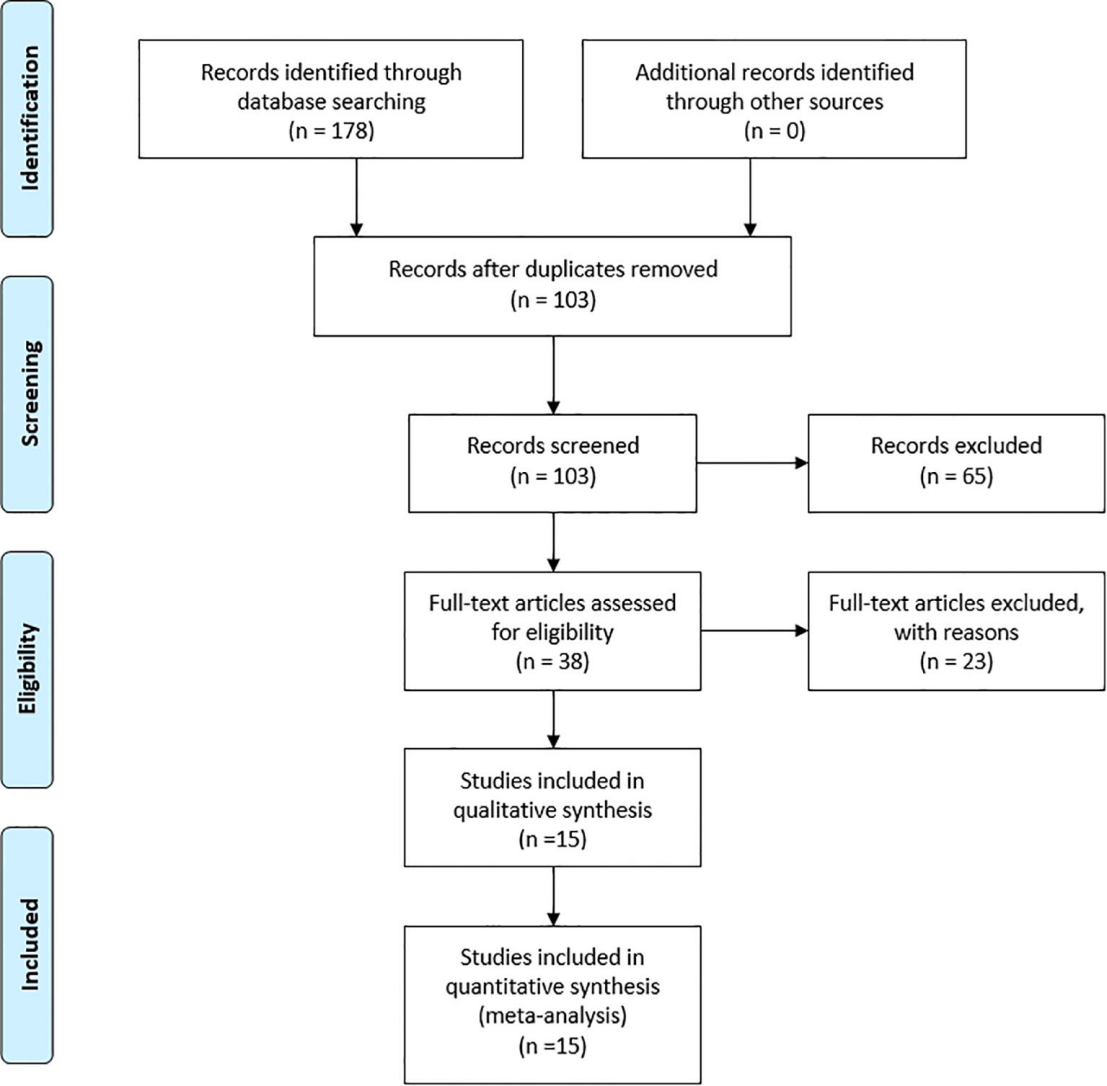
Flow diagram.

The study selection process is shown in **Figure 1**. 178 records were retrieved from four databases (PubMed, Embase, Web of Science, and CNKI) from the inception to April 1, 2024. After excluding duplicates, we screened 103 articles based on their titles and abstracts. The full text of 38 articles was then retrieved for further evaluation. After the full-text evaluation, we excluded 12 articles that met exclusion criteria such as meta-analysis, review, or lack of complete genotype data. 26 articles that met the inclusion criteria were included in the meta-analysis. The literature that can be merged with data was retained, and finally, 15 studies were included.

### Studies characteristics

Among the 15 articles, a total of 2825 patients of NPC and 3,752 healthy controls were included in this meta-analysis. There were 5 studies (31–35) on IL-8 251 A>T polymorphism, 5 studies (16, 36–39) on IL-10 1082A/G polymorphism, 3 studies (16, 38, 39) on IL-10 819 T>C polymorphism, 3 studies (37–39) on IL-10 592 C>A polymorphism, 6 studies (10, 24, 28, 38, 40, 41) on IL-18 607 C>A and IL-18 137 G>C polymorphism. 10 studies (10, 16, 24, 32–35, 39–41) involved Asian populations, 4 studies (28, 31, 36, 37) involved African populations and 1 study (38) involved European populations. In terms of genotyping methods, allele-specific PCR (AS-PCR) was used in 3 studies (31, 36, 38), and polymerase chain reaction-restriction fragment length polymorphism (PCR-RFLP) was used in the remaining 12 studies (10, 16, 24, 28, 32–35, 37, 39–41). According to the NOS assessment, all studies were of high quality. Summary genotype counts for SNP variants and study characteristics are shown in **Table 1**. The results of the meta-analysis are shown in **Table 2**.

**Table 1:** Characteristics of the included studies in the meta-analysis.

First author	Year	Ethnicity	SNP	Age Case Control		Gender N(male/female) Case Control		Case	Control	Genotype distribution Case Control TT AA TA TT AA TA							HWE (p-value)	Genotyp methods	NOS Score
Nasra (31)	2007	African	IL-8 251	42± 16	40 ± 14	115/45	111/58	160	169	49	37	74	75	23	71		0.35	AS-PCR	9
Pan (33)	2020	Asian	IL-8 251	49.39.2	48.7 ± 10.3	122/46	168/64	168	232	76	26	66	65	55	115		0.77	PCR-RFLP	9
Wei (42)	2007	Asian	IL-8 251	49.4 ± 9.5	48.2 ± 10.3	204/76	192/98	280	290	89	54	137	126	42	122		0.17	PCR-RFLP	9
Huang (32)	2018	Asian	IL-8 251	49.3	48.7	128/48	256/96	176	352	80	27	69	121	73	158		0.11	PCR-RFLP	9
Tsai (34)	2007	Asian	IL-8 251	/	/	/	/	105	109	42	11	52	39	17	53		0.89	PCR-RFLP	7
First author	Year	Ethnicity	SNP	Age (mean+SD) year Case Control		Gender N(male/female)Case Control		Case	Control	Genotype distribution Case Control GG AA AG GG AA AG							HWE (p-value)	Genotyp methods	NOS Score
Tsai (16)	2013	Asian	IL10 -1082	48.2	48.9	128/48	379/143	176	522	10	117	49	11	419	92		0.05	PCR-RFLP	9
Farhat (28)	2008	African	IL10 -1082	41.9 ± 15.7	40.4 ± 14.8	116/44	149/48	160	197	22	58	80	26	70	60		0.04	AS-PCR	9
Pratesi (38)	2005	European	IL10 -1082	/	/	70/19	100/30	89	130	19	29	41	26	46	58		0.33	PCR-RFLP	7
Wei (42)	2007	Asian	IL10 -1082	48.7 ± 9.8	47.9 ± 10.1	143/55	139/71	198	210	14	123	61	5	167	38		0.16	PCR-RFLP	9
Moumad (37)	2022	African	IL10 -1082	40.8	41.06	295/145	298/137	384	375	64	182	138	50	169	156		0.15	PCR-RFLP	9
First author	Year	Ethnicity	SNP	Age (mean+SD) year Case Control		Gender N(male/female) Case Control		Case	Control	Genotype distribution Case Control CC TT CT CC TT CT							HWE (p-value)	Genotyp methods	NOS Score
Tsai (16)	2013	Asian	IL10 -819	48.2	48.9	128/48	379/143	176	522	19	88	69	52	285	185		<0.01	PCR-RFLP	9
Pratesi (38)	2005	European	IL10 -819	/	/	70/19	100/30	89	130	48	5	36	70	6	54		0.26	AS-PCR	7
Wei (42)	2007	Asian	IL10 -819	48.7 ± 9.8	47.9 ± 10.1	143/55	139/71	198	210	35	82	81	24	94	92		0.84	PCR-RFLP	9
First author	Year	Ethnicity	SNP	Age (mean+SD) year Case Control		Gender N(male/female) Case Control		Case	Control	Genotype distribution Case Control CC AA AC CC AA AC							HWE (p-value)	Genotyp methods	NOS Score
Pratesi (38)	2005	European	IL-10 592	/	/	70/19	100/30	89	130	48	5	36	70	6	54		0.26	AS-PCR	7
Wei (42)	2007	Asian	IL-10 592	48.7 ± 9.8	47.9 ± 10.1	143/55	139/71	198	210	35	82	81	24	94	92		0.84	PCR-RFLP	9
Moumad (37)	2022	African	IL-10 592	40.8	41.06	295/145	298/137	384	375	215	28	141	195	35	145		0.29	PCR-RFLP	9
Farhat (28)	2008	African	IL-18 607	41.97 ± 16	42.09 ± 15.55	116/47	116/48	163	164	41	28	94	53	34	77		0.54	PCR-RFLP	9
Pratesi (38)	2005	European	IL-18 607	/	/	70/19	100/30	89	130	26	21	42	43	23	64		0.92	AS-PCR	7
Nong (24)	2009	Asian	IL-18 607	48.6 ± 8.9	47.7 ± 9.1	176/74	179/91	250	270	47	71	132	69	68	133		0.81	PCR-RFLP	9
Huang (41)	2018	Asian	IL-18 607	49.3	48.7	128/48	256/96	176	352	30	59	87	88	86	178		0.83	PCR-RFLP	9
Pan (10)	2013	Asian	IL-18 607	48 ± 8	47 ± 8	135/55	140/60	190	200	40	53	97	56	51	93		0.33	PCR-RFLP	9
Du (40)	2012	Asian	IL-18 607	49.8 ± 10.5	49.8 ± 10.5	/	/	150	180	36	34	80	47	40	93		0.64	PCR-RFLP	8
First author	Year	Ethnicity	SNP	Age (mean+SD) year Case Control		Gender N(male/female) Case Control		Case	Control	Genotype distribution Case Control GG CC GC GG CC GC							HWE (p-value)	Genotyp methods	NOS Score
Farhat (28)	2008	African	IL-18 137	41.97 ± 16	42.09 ± 15.55	116/47	116/48	163	164	75	15	73	83	13		68	0.86	PCR-RFLP	9
Pratesi (38)	2005	European	IL-18 137	/	/	70/19	100/30	89	130	43	7	39	72	5		53	0.19	AS-PCR	7
Nong (24)	2009	Asian	IL-18 137	48.6 ± 8.9	47.7 ± 9.1	176/74	179/91	250	270	140	22	88	189	11		70	0.19	PCR-RFLP	9
Huang (41)	2018	Asian	IL-18 137	49.3	48.7	128/48	256/96	176	352	133	5	38	281	6		65	0.35	PCR-RFLP	9
Pan (10)	2013	Asian	IL-18 137	48 ± 8	47 ± 8	135/55	140/60	190	200	102	14	74	139	9		52	0.18	PCR-RFLP	9
Du (40)	2012	Asian	IL-18 137	49.8 ± 10.5	49.8 ± 10.5	/	/	150	180	88	11	51	131	6		43	0.32	PCR-RFLP	8

SNP, single nucleotide polymorphisms; NOS, The Newcastle-Ottawa Scale; HWE, Hardy–Weinberg equilibrium; PCR-RFLP, polymerase chain reaction-restriction fragment length polymorphism; AS-PCR, allele-specific PCR.

**Table 2:** Meta-analysis results.

	n	Case/control	OR	95% CI	P	I^2^ (%)	OR	95% CI	P	I^2^ (%)	OR	95% CI	P	I^2^ (%)	OR	95% CI	P	I^2^ (%)	OR	95% CI	P	I^2^ (%)
			Allele model				Dominant model				Recessive model				Heterozygote model				Homozygote model			
IL-8 251A>T Total	5	889/1152	0.89	0.58-1.37	0.60	91	-0.01	-0.14-0.11	0.82	88	0.95	0.6-1.51	0.83	72	0.95	0.58-1.53	0.82	83	0.91	0.45-1.88	0.81	86
Ethnicity Asian	4	729/983	1.05	0.72-1.53	0.81	85	1.12	0.66-1.92	0.67	85	1.08	0.65-1.79	0.78	70	1.12	0.7-1.77	0.64	76	1.13	0.54-2.39	0.75	84
IL-10 1082A>G Total	5	1007/1434	1.35	0.95-1.9	0.09	83	1.48	0.99-2.19	0.05	78	1.40	0.91-2.17	0.12	52	1.43	0.95-2.15	0.09	77	1.57	0.98-2.52	0.06	54
Ethnicity Asian	2	374/732	2.10	1.64-2.69	0.000	0	2.19	1.64-2.92	0.000	0	2.95	1.50-5.77	0.002	0	2.02	1.49-2.75	0.000	0	3.51	1.78-6.90	0.000	0
IL-10 819T>C Total	3	463/862	0.87	0.73-1.04	0.13	0	0.82	0.6-1.13	0.22	0	0.86	0.67-1.11	0.25	0	0.85	0.61-1.19	0.34	0	0.75	0.51-1.11	0.15	0
Ethnicity Asian	2	374/732	0.85	0.70-1.03	0.09	0	0.74	0.50-1.09	0.13	8	0.85	0.66-1.10	0.21	0	0.79	0.52-1.19	0.26	33	0.71	0.47-1.08	0.11	0
IL-10 592C>A Total	3	671/715	0.86	0.73-1.02	0.08	0	0.83	0.66-1.04	0.10	0	0.85	0.63-1.15	0.30	0	0.84	0.66-1.08	0.17	0	0.7	0.48-1.03	0.07	0
IL-18 607C>A Total	6	1018/1296	1.21	1.07-1.36	0.002	0	1.24	0.97-1.58	0.02	31	1.14	0.92-1.40	0.18	12	1.37	1.12-1.68	0.003	0	1.46	1.15-1.85	0.002	0
Ethnicity Asian	4	766/1002	1.23	1.08-1.41	0.003	0	1.19	0.83-1.71	0.34	57	1.18	0.95-1.46	0.14	14	1.37	1.08-1.75	0.01	0	1.53	1.17-2.02	0.002	0
IL-18 137G>C Total	6	1018/1296	1.50	1.30-1.73	0.000	13	1.58	1.33-1.89	0.000	2	1.79	1.24-2.60	0.002	0	1.51	1.26-1.81	0.000	0	2.07	1.42-3.02	0.000	0
Ethnicity Asian	4	766/1002	1.65	1.38-1.96	0.000	0	1.73	1.41-2.12	0.000	0	2.01	1.28-3.17	0.003	0	1.64	1.32-2.04	0.000	0	2.39	1.51-3.78	0.000	0

OR, odds ratio; CI, confidence interval.

### Association of the IL-8 polymorphisms with the risk of NPC

For the IL-8 251A>T polymorphism, a total of 5 articles with 889 NPC patients and 1152 healthy controls were included. The results showed that there were no significant associations between IL-8 251 A>T polymorphisms and the risk of NPC. Subgroup analysis by ethnicity showed that there were also no significant associations between IL-8 251 A>T polymorphisms and the risk of NPC.

### Association of the IL-10 polymorphisms with the risk of NPC

**Figure 2:**
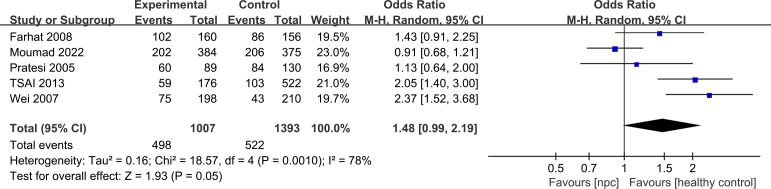
Forest plots of IL-10 1082A>G polymorphism and nasopharyngeal carcinoma risk in dominant model.

**Figure 3:**
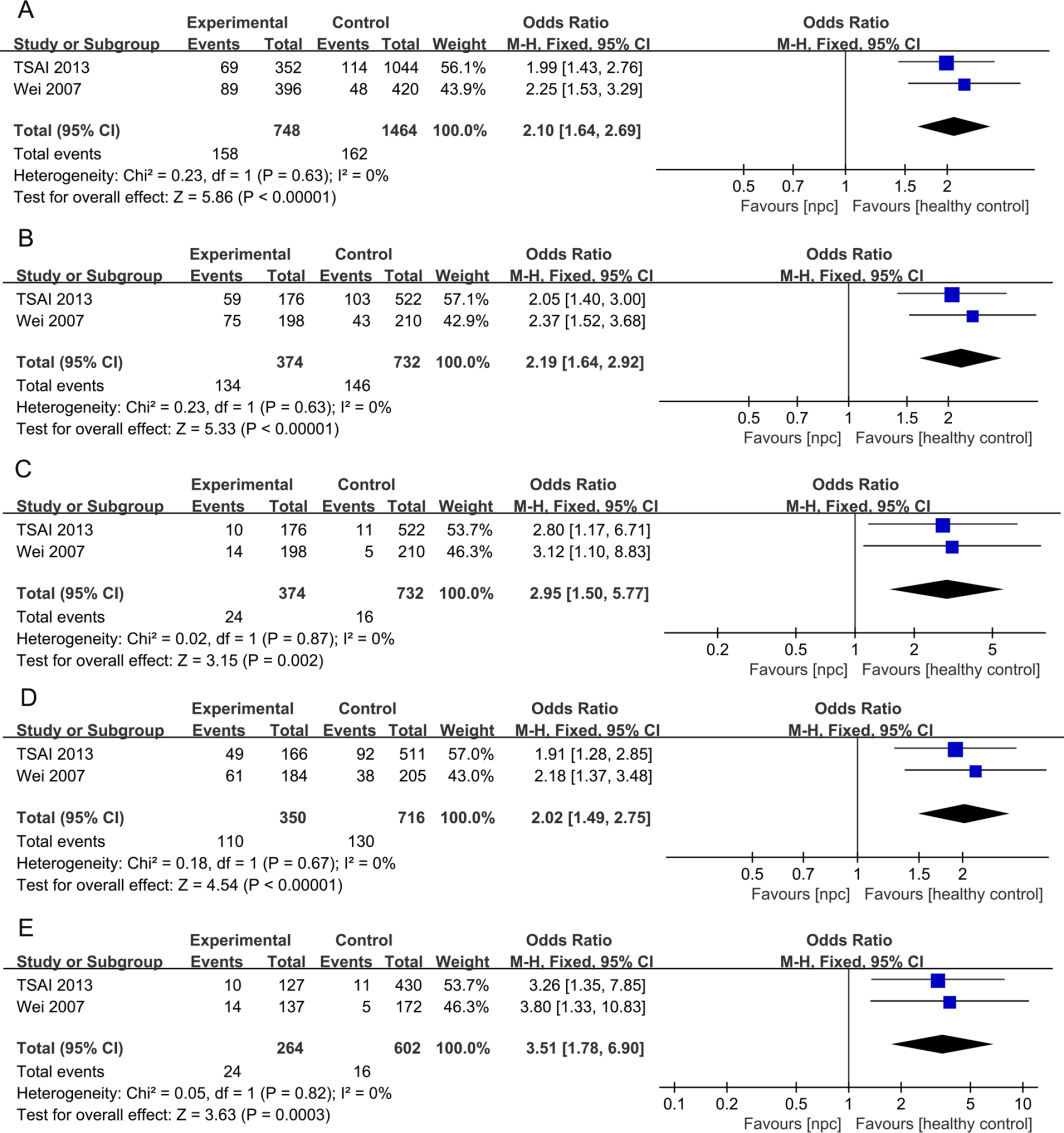
Forest plots of IL-10 1082A>G polymorphism and nasopharyngeal carcinoma risk-stratified according to ethnicity. **(A)** Allele model; **(B)** Dominant model; **(C)** Recessive model; **(D)** Heterozygote model; **(E)** Homozygote model.

For the IL-10 1082A>G polymorphism, a total of 5 articles with 1007 NPC patients and 1434 healthy controls were included. The results showed that significant results were only found in the Dominant model (OR=1.48, 95% CI = 0.99-2.19, p=0.05, I^2^ = 78%, **Figure 2**), and there were no significant results in the other four models. However, after subgroup analysis, IL-10 1082A>G was found to be significantly associated with the risk of NPC in Asian populations(Allele model: OR=2.10, 95% CI = 1.64-2.69, p<0.00001, I^2^ = 0%, **Figure 3A**, Dominant model: OR=2.19, 95% CI = 1.64-2.92, p<0.00001, I^2^ = 0%, **Figure 3B**, Recessive model: OR=2.95, 95% CI = 1.50-5.77, p=0.002, I^2^ = 0%, **Figure 3C**, Heterozygote model OR=2.02, 95% CI = 1.49-2.75, p<0.00001, I^2^ = 0%, **Figure 3D**, Homozygote model OR=3.51, 95% CI = 1.78-6.90, p=0.0003, I^2^ = 0%, **Figure 3E**).

For the IL-10 819T>C polymorphism, a total of 3 articles with 463 NPC patients and 862 healthy controls were included. The results showed that there were no significant associations between IL-10 819T>C polymorphisms and The risk of NPC. Subgroup analysis by ethnicity showed that there were also no significant associations between IL-10 819T>C polymorphisms and the risk of NPC.

For the IL-10 592C>A polymorphism, a total of 3 articles with 671 NPC patients and 715 healthy controls were included. The results showed that there were no significant associations between IL-10 592C>A polymorphisms and the risk of NPC.

### Association of the IL-18 polymorphisms with the risk of NPC

**Figure 4:**
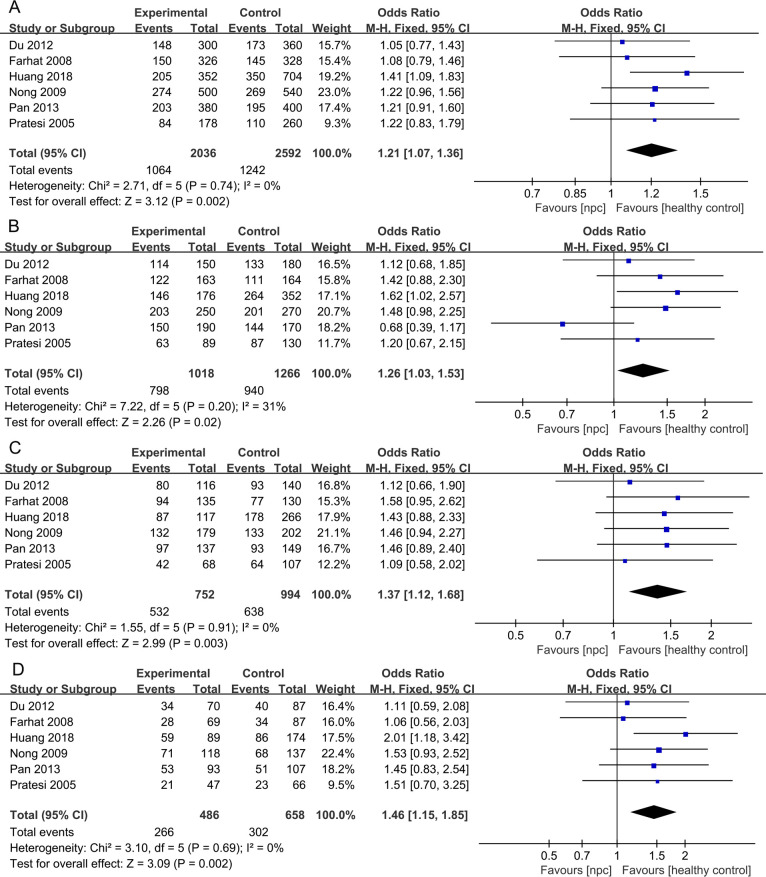
Forest plots of IL-18 137G>C polymorphism and nasopharyngeal carcinoma risk. **(A)** Allele model; **(B)** Dominant model; **(C)** Heterozygote model; **(D)** Homozygote model.

**Figure 5:**
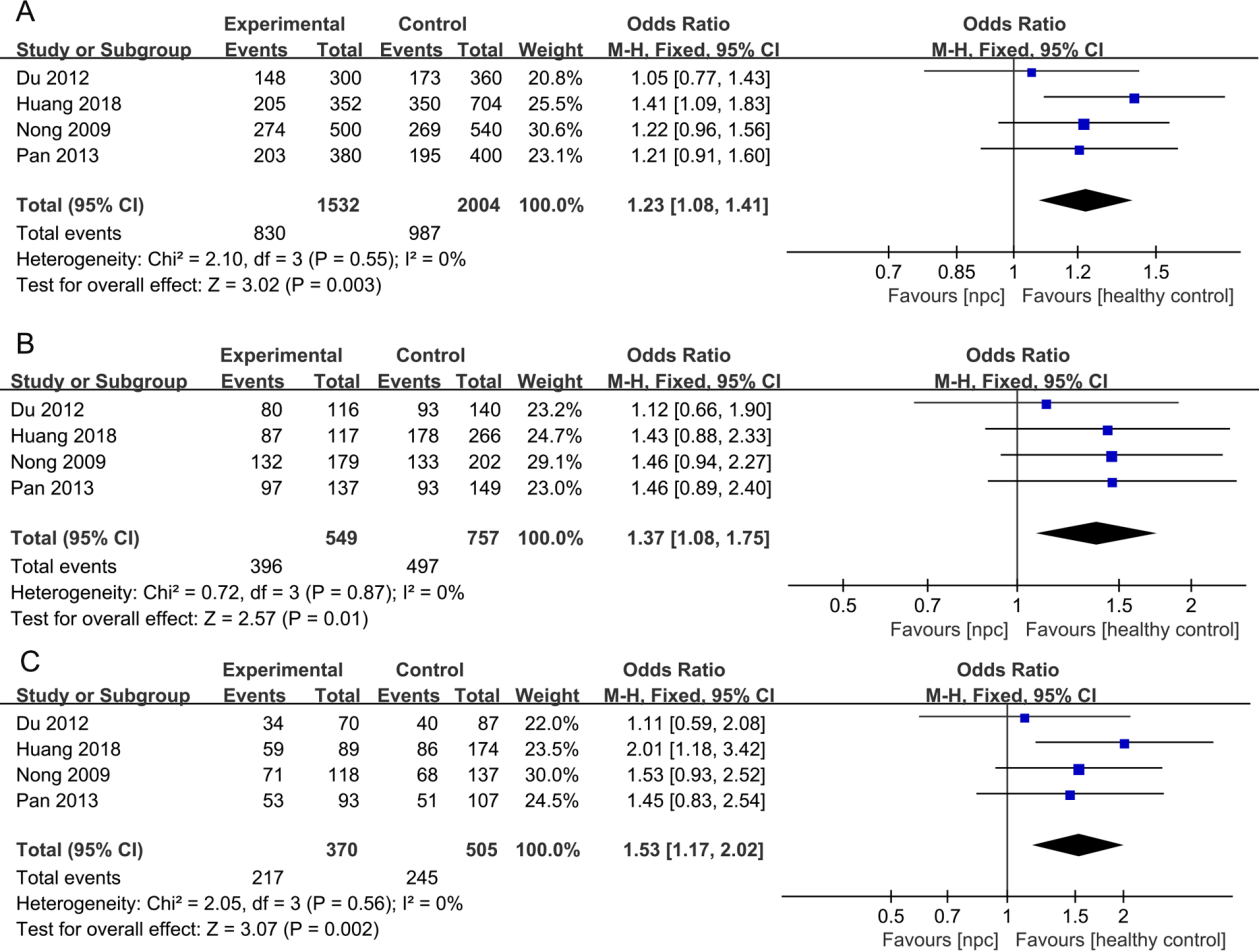
Forest plots of IL- 18 607C>A polymorphism and nasopharyngeal carcinoma risk stratified according to ethnicity. **(A)** Allele model; **(B)** Heterozygote model; **(C)** Homozygote model.

For the IL- 18 607C>A polymorphism, a total of 6 articles with 1018 NPC patients and 1296 healthy controls were included. The results showed that significant results were found in four models (Allele model: OR=1.21, 95% CI = 1.07-1.36, p=0.002, I^2^ = 0%, **Figure 4A**, Dominant model: OR=1.26, 95% CI = 1.03-1.53, p=0.02, I^2^ = 31%, **Figure 4B**, Heterozygote model OR=1.37, 95% CI = 1.12-1.68, p=0.003, I^2^ = 0%, **Figure 4C**, Homozygote model OR=1.46, 95% CI = 1.15-1.85, p=0.002, I^2^ = 0%, **Figure 4D**), but there were no significant results in Recessive model. Subgroup analysis by ethnicity showed that in Asian populations, IL- 18 607C>A was found to be significantly associated with the risk of NPC in three models (Allele model: OR=1.23, 95% CI = 1.08-1.41, p=0.003, I^2^ = 0%, **Figure 5A**, Heterozygote model OR=1.37, 95% CI = 1.08-1.75, p=0.01, I^2^ = 0%, **Figure 5B**, Homozygote model OR=1.53, 95% CI = 1.17-2.02, p=0.002, I^2^ = 0%, **Figure 5C**), but there were no significant results in Dominant model and Recessive model.

**Figure 6:**
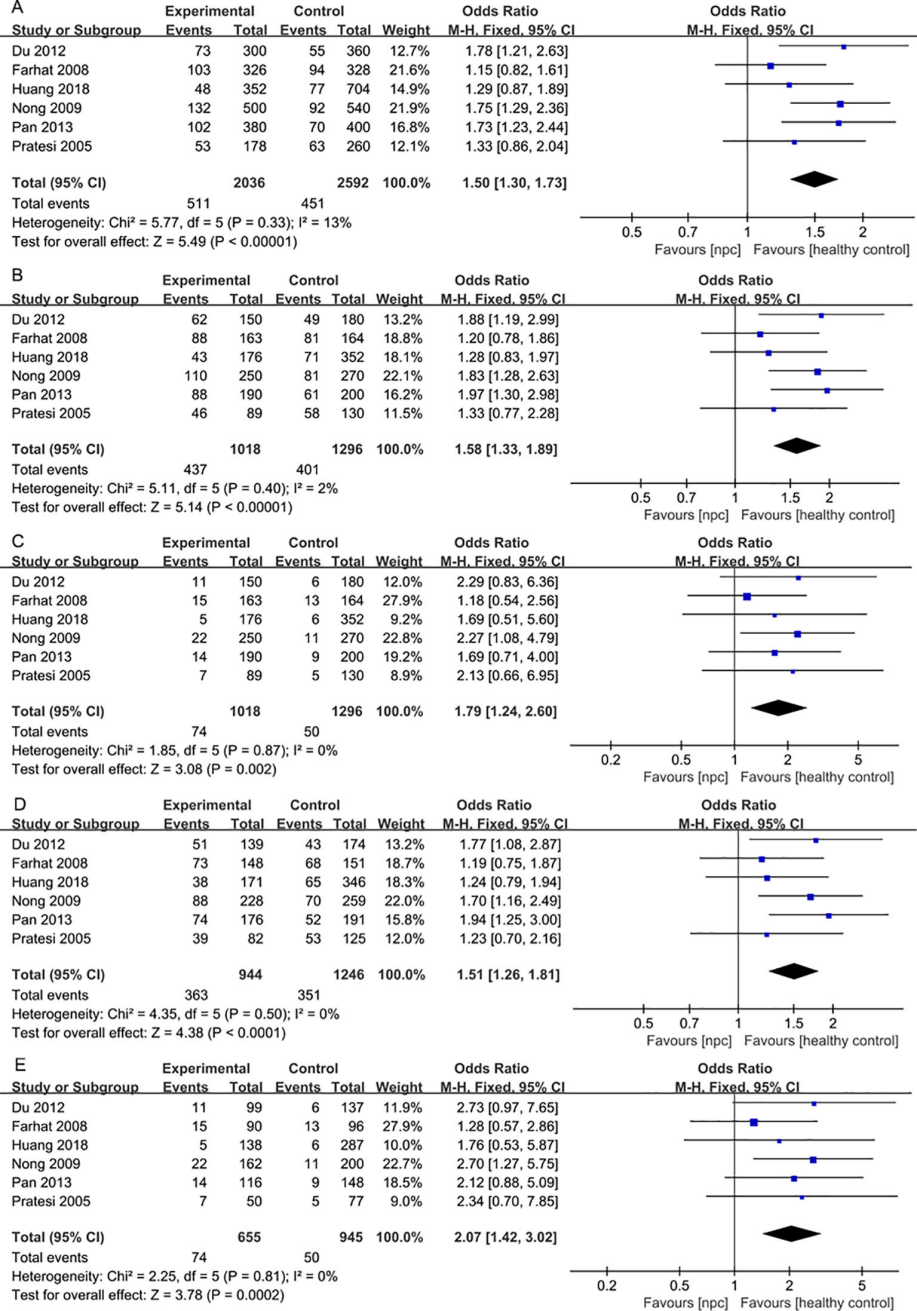
Forest plots of IL-18 137G>C polymorphism and nasopharyngeal carcinoma risk. **(A)** Allele model; **(B)** Dominant model; **(C)** Recessive model; **(D)**Heterozygote model; **(E)** Homozygote model.

**Figure 7:**
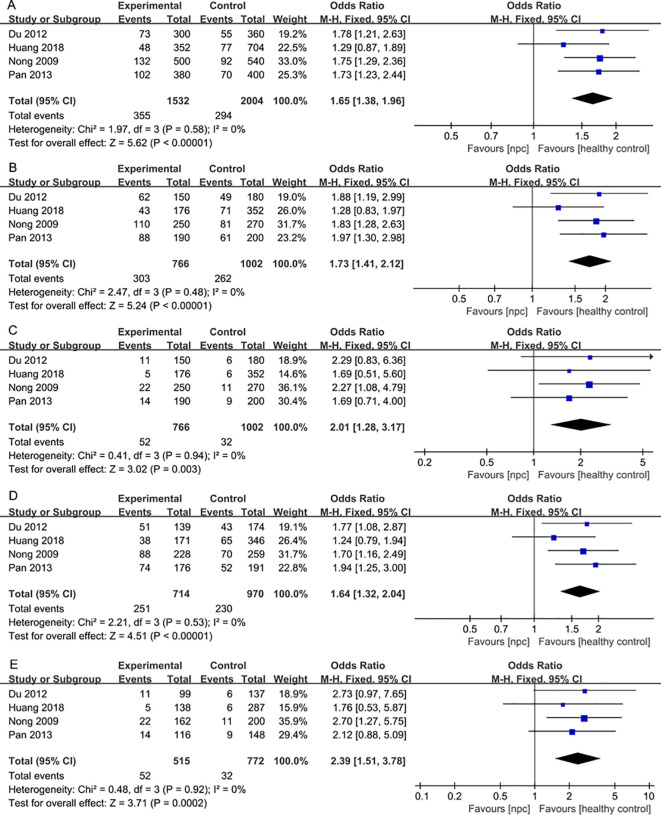
Forest plots of IL-18 137G>C polymorphism and nasopharyngeal carcinoma risk stratified according to ethnicity. **(A)** Allele model; **(B)** Dominant model; **(C)** Recessive model; **(D)** Heterozygote model; **(E)** Homozygote model.

For the IL-18 137G>C polymorphism, a total of 6 articles with 1018 NPC patients and 1296 healthy controls were included. IL-18 137G>C was found to be significantly associated with the risk of NPC (Allele model: OR=1.50, 95% CI = 1.30-1.73, p<0.00001, I^2^ = 13%, **Figure 6A**, Dominant model: OR=1.58, 95% CI = 1.33-1.89, p<0.00001, I^2^ = 2%, **Figure 6B**, Recessive model: OR=1.79, 95% CI = 1.24-2.60, p=0.002, I^2^ = 0%, **Figure 6C**, Heterozygote model OR=1.51, 95% CI = 1.26-1.81, p<0.00001, I^2^ = 0%, **Figure 6D**, Homozygote model OR=2.07, 95% CI = 1.42-3.02, p=0.0002, I^2^ = 0%, **Figure 6E**). Subgroup analysis by ethnicity showed that IL-18 137G>C was significantly associated with the risk of NPC in Asian populations (Allele model: OR=1.65, 95% CI = 1.38-1.96, p<0.00001, I^2^ = 0%, **Figure 7A**, Dominant model: OR=1.73, 95% CI = 1.41-2.12, p<0.00001, I^2^ = 0%, **Figure 7B**, Recessive model: OR=2.01, 95% CI = 1.28-3.17, p=0.003, I^2^ = 0%, **Figure 7C**, Heterozygote model OR=1.64, 95% CI = 1.32-2.04, p<0.00001, I^2^ = 0%, **Figure 7D**, Homozygote model OR=2.39, 95% CI = 1.51-3.78, p=0.0002, I^2^ = 0%, **Figure 7E**).

### Sensitivity analysis

Some of the results showed high heterogeneity after pooling, and the reason for the high heterogeneity was not found by subgroup analysis. We performed a sensitivity analysis to investigate the influence of each study on the overall pooled results. There were no obvious changes after systematically excluding each of the studies, showing their stability, so we cannot determine which studies had a significant effect on the results.

## Discussion

NPC is a rare malignant epithelial tumor that is common in southern and southeastern China, the global incidence of NPC is 1.5 cases per 100,000 person-years, with East Asia accounting for about 49.39% of all NPC cases, and Southeastern Asia following with 27.55% of the total cases (3). There is strong evidence showing that the occurrence of NPC is closely associated with EBV virus infection (43). In addition, there is also increasing evidence showing that inflammatory cytokines also play an important role in its pathogenesis (44, 45). In 1863, Virchow noticed the presence of white blood cells in tumor tissue and was the first to suggest that inflammation was associated with tumor formation (46). Many studies have shown that NPC is characterized by a high level of leukocyte infiltration in tumor cells (47, 48).

Large-sample epidemiological studies of genetic polymorphisms can provide deep insights into the associations between candidate genes and diseases, which has become a crucial determinant for disease susceptibility and severity (49, 50). Our meta-analysis included 15 high-quality case-control studies, encompassing 2825 NPC patients and 3752 healthy controls. We evaluated a comprehensive meta-analysis of IL-8 251 A>T, IL-10 (1082A/G, 819 T>C, and 592 C>A), and IL-18 (607 C>A and 137 G>C) polymorphisms and their association with NPC susceptibility.

IL-10 is primarily produced by macrophages and T lymphocytes and is an important anti-inflammatory and immunosuppressive cytokine that acts by down-regulating the expression of T helper 1 (Th1) cytokines and co-stimulatory molecules (51). There are many genetic variations of the IL-10 gene, and the three most studied SNPs in the promoter region (-1082 (G/A), -819 (C/T), and -592 (C/A)) have been shown to alter IL-10 mRNA and protein levels, thereby influencing the progression of diseases. Studies indicate that compared to the control group, the high expression of the -1082G allele in patients is associated with diseases such as lupus, non-small cell lung cancer, cervical cancer, and oral cancer, and promotes the development of the pathological processes of these diseases (43, 52, 53). It is hypothesized that IL-10 may help tumor cells evade immune surveillance and potentially promote tumor growth (54). In Fujieda’s study (55), the expression of IL-10 in primary NPC was investigated by immunohistochemical methods, and the results showed that IL-10 expression was significant as an independent prognostic indicator of overall survival. It serves as a prognostic factor for NPC and is valuable in selecting appropriate aggressive treatments for NPC patients. Another study showed that IL-10 promotes cell proliferation and cell cycle progression via the JAK2/STAT3 signaling pathway in NPC (56).

Our meta-analysis of the IL-10-1082 A/G polymorphism revealed that only the dominant model showed a significant association with the risk of NPC in the overall population. We conducted subgroup analyses stratified by geographic location (Asian and non-Asian), which indicated that there is a significant association between the IL-10-1082 A/G polymorphism and the risk of NPC in Asian populations. Therefore, we believe that different genetic backgrounds and environments among different ethnicities strongly influence the distribution of IL-10 polymorphisms, further influencing genetic risk. Due to the continuous long-distance migration, adaptation to different environments, and interracial mating, the genetic structure of human populations is different (38, 57). Studies have shown that these genetic differences can affect people’s susceptibility to disease, resulting in the presence of specific genetic risk factors in different ethnic groups (7). The differences between race-related SNPs and disease susceptibility are crucial for identifying disease risk factors for each population and for planning responses. Given the critical role of IL-10 in inflammatory responses, tumor development, and metastasis, in combination with previous studies and our meta-analysis, we speculate that the IL-10-1082 A/G polymorphism is associated with the risk of NPC in Asian populations through the modulation of IL-10 expression.

IL-18, a pleiotropic proinflammatory cytokine, is a key cytokine in the immune response (58). IL-18 has been shown to enhance IFN-γ production by T and NK cells, promote cell death, and inhibit tumor progression (44). However, the role of IL-18 in cancer is controversial. Other studies have shown that tumor cells can evade immune recognition and promote tumor cell proliferation and metastasis through IL-18. Ma et al. (12) showed that the expression of IL-18 was significantly different between breast cancer and fibroadenoma tissues by immunohistochemistry, and the expression of IL-18 was positive in breast cancer tissues. Another study (59) showed the relationship between the levels of myeloid-derived suppressor cells (MDSCs) and IL-18 expression in osteosarcoma tumor models. The results suggest that blocking IL-18 may reduce the accumulation and function of MDSCs, thereby enhancing the efficacy of anti-PD-1 therapy in osteosarcoma patients. A recent multicenter, randomized, phase 3 trial conducted by Liu et al. (60) demonstrated that a PD-1 monoclonal antibody therapy, sintilimab, can improve progression-free survival rates in NPC patients. Combining these two studies, we hypothesize that the combined use of sintilimab after blocking IL-18 may be more effective in reducing disease progression and improving patient survival.

Studies have demonstrated that the IL-18 gene -137G/C polymorphism is strongly associated with colorectal cancer, esophageal cancer, and other diseases (61, 62). The polymorphism of IL-18 may affect the gene expression of IL-18 (63), therefore, it is important to investigate the genetic polymorphisms on IL-18 and NPC susceptibility. Previous clinical studies of IL-18 polymorphisms and cancer risk have yielded controversial results. Therefore, we performed this meta-analysis to determine the exact association of the IL-18 polymorphism with the risk of NPC.

In addition to IL-8, IL-10, and IL-18, the included studies also addressed other cytokines, such as IL-1, IL-2, IL-12, IL-13, and IL-16. However, due to the limited number of related studies, we were unable to combine the results of these cytokines for a meta-analysis. Nevertheless, the importance of these cytokines in the immune response should not be overlooked. For example, IL-1 is a pro-inflammatory cytokine that plays a role in the development and progression of various cancers, particularly in the formation of the inflammatory microenvironment (64); IL-2 is closely associated with T-cell proliferation and immune responses, potentially playing an important role in tumor immune evasion (25); IL-16 is involved in the recruitment and activation of T-cell, contributing to immune responses (65). In the future, large-scale and multi-center studies could further investigate the specific roles of these cytokines in NPC, especially in the complex interactions within the cytokine network and the immune microenvironment, and potentially identify new targets for immunotherapy.

Our study has several limitations. First, there is a lack of uniform standards among the included studies, leading to inconsistencies in some factors such as age, diet, and lifestyle. Second, there was significant heterogeneity in some of the models. Several factors could explain this heterogeneity, including differences in the size of control groups, differences in population characteristics such as ethnicity, differences in genotyping methods, and differences in study design. Although subgroup analyses were conducted to elucidate the sources of heterogeneity, it was difficult to identify all potential contributors. Third, the meta-analysis was based on a small sample size, which may increase the risk of random error. Further research is needed to confirm and strengthen the findings regarding the association between cytokine gene polymorphisms and NPC susceptibility, with larger sample sizes and higher-quality studies. Nonetheless, the study provides valuable insights with clinical relevance, offering practical applications that can enhance clinical decision-making for NPC. First, this meta-analysis has revealed that IL-10 and IL-18 can be used as markers of genetic susceptibility to NPC. Based on this finding, genetic testing tools can be developed. Through early detection, potential high-risk individuals can be screened out, and early intervention and regular monitoring can be carried out in advance. Second, IL-10 and IL-18 play an important role in the immune microenvironment of tumors, so we can improve the effectiveness of immunotherapy by adjusting the levels of IL-10 and IL-18. Third, it provides new ideas for the development of new immunotherapy strategies. By regulating these factors, the patient’s immune response may be enhanced and tumor growth may be inhibited.

## Conclusion

In conclusion, our study showed that the IL-10 1082A>G polymorphism was significantly associated with the risk of higher-grade NPC in the Asian population. IL-18 607C>A and IL-18 137G>C polymorphisms were significantly associated with the overall risk of NPC. However, given the limited sample size and unknown factors, future studies with larger sample sizes, multicenter settings, and longer follow-up periods are warranted to draw more reliable conclusions.

## Data Availability

The original contributions presented in the study are included in the article/supplementary material, further inquiries can be directed to the corresponding author/s.
